# Lusutrombopag is effective and safe in patients with chronic liver disease and severe thrombocytopenia: a multicenter retrospective study

**DOI:** 10.1186/s12876-020-01573-9

**Published:** 2020-12-14

**Authors:** Hiroaki Nomoto, Naoki Morimoto, Kouichi Miura, Shunji Watanabe, Yoshinari Takaoka, Hiroshi Maeda, Takahiro Sasaki, Yohei Koyashiki, Hidekazu Kurata, Norikatsu Numao, Norio Isoda, Hironori Yamamoto

**Affiliations:** 1 Department of Medicine, Division of Gastroenterology, Jichi Medical University, 3311-1 Yakushiji, Shimotsuke, 329-0498 Japan; 2 Department of Radiology, Jichi Medical University, Shimotsuke, 329-0498 Japan; 3 Department of Gastroenterology, Tochigi Medical Center Shimotsuga, Tochigi, 329-4498 Japan; 4 Department of Gastroenterology, Haga Red Cross Hospital, Mooka, 321-4308 Japan

**Keywords:** Liver cirrhosis, Thrombocytopenia, Thrombopoietin receptor agonist, Platelet transfusion

## Abstract

**Background:**

Chronic liver disease (CLD) is often complicated by severe thrombocytopenia (platelet count < 50,000/µL). Platelet transfusion has been a gold standard for increasing the platelet count to prevent hemorrhagic events in such patients. Lusutrombopag, a thrombopoietin receptor agonist, can increase the platelet count in such patients when invasive procedures are scheduled. Former studies on lusutrombopag included patients with a platelet count of > 50,000/µL at baseline: the proportions of patients who did not require platelet transfusion were 84–96%, which might be overestimated.

**Methods:**

The efficacy and safety of lusutrombopag were retrospectively investigated in CLD patients with platelet count of < 50,000/µL, a criterion for platelet transfusion, in real-world settings. We examined the proportion of patients who did not require platelet transfusion in 31 CLD patients, which exceeded a minimum required sample size (21 patients) calculated by 80% power at a significance level of 5%. Lusutrombopag, 3 mg once daily, was administered 8–18 days before scheduled invasive procedures.

**Results:**

Among 31 patients who received lusutrombopag, 23 patients (74.2%) patients showed a platelet count of ≥ 50,000/µL (Group A) and did not require platelet transfusion. The remaining 8 patients (25.8%) did not reached platelet ≥ 50,000/µL (Group B). The means of platelet increase were 38,000/µL and 12,000/µL in groups A and B, respectively. A low platelet count at baseline was a characteristic of patients in group B. Among 13 patients who repeatedly used lusutrombopag, lusutrombopag significantly increased the platelet count as the initial treatment. When all repeated uses of lusutrombopag were counted among these 13 patients, platelet transfusion was not required in 82.1% (23/28) of treatments. Although one patient showed portal thrombosis after lusutrombopag treatment, the thrombosis was disappeared by anticoagulant treatment for 35 days. The degree of platelet increase with lusutrombopag was larger than that in their previous platelet transfusion.

**Conclusions:**

The proportion of patients who did not require platelet transfusion was 74.2%, which is smaller than that in former studies which included CLD patients with a platelet count of > 50,000/µL. However, lusutrombopag is effective and safe for CLD patients with a platelet count of < 50,000/µL.

## Background

Chronic liver disease (CLD) is frequently complicated by thrombocytopenia [1]. Indeed, 10% of patients with cirrhosis show platelet counts of < 50,000/µL [2], a criterion for platelet transfusion when invasive procedures are required [3]. In fact, these patients often have hepatocellular carcinoma and gastrointestinal varices, which require invasive therapeutic procedures, including radiofrequency ablation and ligation/sclerotherapy, respectively. These invasive procedures are associated with an increased risk of bleeding events [4]. Thus, it is necessary to increase the platelet count to prevent hemorrhagic events.

Platelet transfusion has been a gold standard to increase platelet count. However, platelet transfusion is associated with several problems, including the risk of unknown infection, allergic reaction and a shortage of donors [5]. In addition, repeated platelet transfusion may induce refractoriness to subsequent platelet transfusion. Furthermore, the efficacy of platelet transfusion is likely to be limited [1]. Thus, alternative methods to platelet transfusion are required to resolve such problems.

In the USA, FDA approved two thrombopoietin receptor agonists, avatrombopag and lusutrombopag, in 2018[6]. These thrombopoietin receptor agonists showed favorable results to increase platelet count in clinical trials [7–9]. In Japan, lusutrombopag was approved for CLD patients with thrombocytopenia in 2015[10]. Japanese real-world data demonstrated that 84–96% of patients who received lusutrombopag treatment did not require platelet transfusion before their scheduled invasive procedure [11–13]. In addition, the increase in the platelet count achieved by lusutrombopag was superior to that did by platelet transfusion [14]. Furthermore, in patients who received lusutrombopag repeatedly, the increase in the platelet count was similar to that in the initial treatment [15, 16]. Adverse events related to lusutrombopag were limited. Thus, lusutrombopag is now one of the choices of treatment for increasing the platelet count in CLD patients with thrombocytopenia.

Although lusutrombopag showed favorable effects in CLD patients with thrombocytopenia, the real-world data on lusutrombopag included patients with a platelet count of > 50,000/µL at baseline [11–13]; thus, some of these patients would not have required platelet transfusion [3]. We therefore investigated the efficacy and safety of lusutrombopag in the real-world among patients with platelet counts of < 50,000/µL, which is generally accepted as a criterion for platelet transfusion.

## Methods

### Patients

We performed a multicenter retrospective study from April 2016 to November 2020. CLD patients with severe thrombocytopenia (< 50,000/µL) were enrolled in the present study. The leading exclusion criteria were portal vein thrombosis, lusutrombopag allergy, splenectomy, partial splenic embolization before lusutrombopag treatment and Child–Pugh class C. Lusutrombopag (3 mg once daily [Mulpleta, Shionogi & Co., Ltd., Osaka, Japan]) was started 8–18 days before a scheduled invasive procedure. On day 5, lusutrombopag was discontinued if the platelet count was ≥ 50,000/µL with an increase of ≥ 20,000/µL. Lusutrombopag was continued 2 more days when platelet count did not reach 50,000/µL. After the administration of lusutrombopag for 5 or 7 days, the platelet count was monitored every 2–4 days from day 5 to the day before the procedures and a couple of time points after the procedures. Maximum platelet count was noted between day 5 and the day before the procedures. We divided the patients into two groups according to the response to lusutrombopag: group A included patients with a platelet counts of ≥ 50,000/µL before the procedures; group B included patients with a platelet count of < 50,000/µL before the procedures. Because platelet count was maintained ≥ 50,000/µL until the day before the procedure once the count reached ≥ 50,000/µL in all cases of group A, the patients in group A did not require platelet transfusion before the invasive treatment. In addition to lusutrombopag treatment, we investigated the efficacy of platelet transfusion. When the platelet count was < 50,000/µL on the previous day of the procedure, 10 units of platelets (> 2 × 10^11^) were transfused just before the invasive procedure. The platelet was also counted on the next day after the procedure in cases of platelet transfusion. Portal vein thrombosis was monitored by abdominal ultrasonography and/or computed tomography (CT). The albumin–bilirubin (ALBI) grade and fibrosis-4 (FIB4) index were calculated according to published formulas [17, 18]. The splenic volume was measured on CT examinations using image processing software (ziostation2, Ziosoft, Tokyo). The spleen volume was automatically calculated after 3-dimensional imaging of the spleen was reconstituted.

### Statistical analyses

A minimum sample size calculated by 80% power at a significance level of 5% was at least 21 patients. The results were analyzed by the chi-squared test, Wilcoxon signed rank test, and univariate and multivariate logistic regression analyses. We performed multivariate analysis when a p-value was < 0.1 in univariate analysis. All statistical analyses were performed using the Stata15 software program (STATA Corporation, College Station, TX, USA). p values of < 0.05 were considered to indicate statistical significance.

## Results

### The efficacy of lusutrombopag

Table 1 shows the characteristics of all 31 patients at the first administration of lusutrombopag. There were 23 (74.2%) and 8 (25.8%) patients in groups A and B, respectively. Group B was characterized by a male predominance, low platelet count at baseline, platelet increase > 20,000/µL, and a high splenic volume. However, there were no significant differences in age, duration of lusutrombopag treatment, history of platelet transfusion, Child–Pugh grade and score, ALBI score, FIB4 index or Mac-2 binding protein glycosylation isomer, and the days from the staring lusutrombopag to the procedure between groups A and B. In addition, etiology is unlikely to contribute to the effect of lusutrombopag.

**Table 1:** The characteristics of patients who received lusutrombopag

	All	Group A	Group B	p-value
n	31	23 (74.2%)	8 (25.8%)	
Male/female	21/10	13/10	8/0	0.0226
Age (years)	64.7 ± 8.8	64.8 ± 9.4	64.3 ± 6.9	0.6838
Lusutrombopag 5/7 days	3/28	3/20	0/8	0.2712
History of platelet transfusion	15/31(48%)	10/23(43%)	5/8(63%)	0.3663
HCV/HBV/NASH/ALC/others	15/2/3/8/3	11/2/2/5/3	4/0/1/3/0	
Child–Pugh A/B	17/14	12/11	5/3	0.6206
Child–Pugh score	6.7 ± 1.3	6.7 ± 1.3	6.6 ± 1.5	0.7443
ALBI	− 2.06 ± 0.62	− 1.95 ± 0.60	− 2.39 ± 0.54	0.0947
FIB-4	13.24 ± 4.55	13.21 ± 5.02	13.33 ± 2.80	0.8923
M2BPGi (COI)	10.03 ± 5.28	10.49 ± 5.62	8.28 ± 3.13	0.3373
TACE/RFA/EVL/EIS/others	14/7/4/1/5	11/6/2/0/4	3/1/2/1/1	
Period until the procedure (day)	12.3 ± 1.9	12.3 ± 1.8	12.4 ± 2.2	0.8728
Platelet count (× 10^4^/µL) < 3.5/3.5–4.5/4.5 <	9/13/9	4/10/9	5/3/0	
Baseline platelet count (× 10^4^/µL)	3.9 ± 0.7	4.2 ± 0.6	3.1 ± 0.6	0.0010
Platelet increase > 2 × 10^4^/µL	22 (71%)	20 (87%)	2 (25%)	0.000802
Splenic volume (mL)	694 ± 321	615 ± 280	922 ± 322	0.0254

*HCV* hepatitis C virus, *HBV* hepatitis B virus, *NASH* non-alcoholic steatohepatitis, *ALC* alcohol, *ALBI* albumin–bilirubin, *FIB-4* fibrosis-4, *M2BPGi* mac2 binding protein glucosylation isomer, *TACE* transcatheter arterial chemoembolization, *RFA* radiofrequency ablation, *EVL* endoscopic variceal ligation, *EIS* endoscopic injection sclerotherapy

**Fig. 1:**
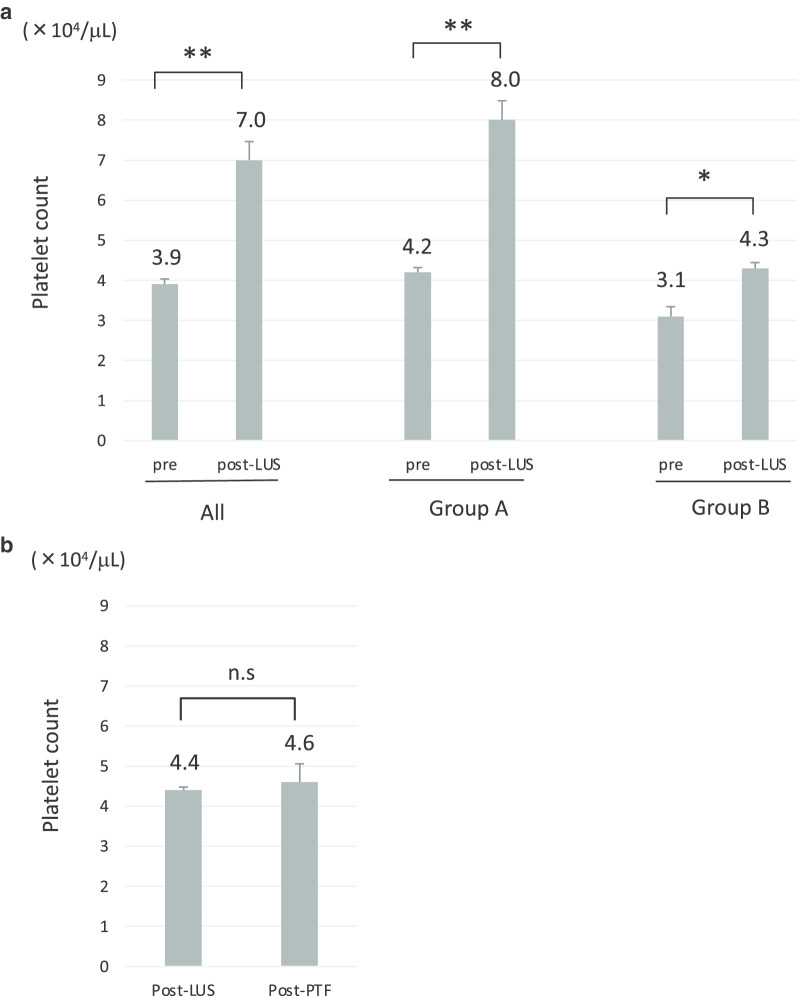
**a** The platelet counts after lusutrombopag treatment in all patients (left panel, n = 31), group A (middle panel, n = 23), and group B (right panel, n = 8). **b** The platelet count in patients who received platelet transfusion after lusutrombopag treatment (n = 7). **p < 0.01, *n.s* not significant, *LUS* lusutrombopag, *PTF* platelet transfusion

Lusutrombopag significantly increased the platelet count in all 31 patients with a mean increase of 31,000/µL (p < 0.01) (Fig. 1a, left panel). The degree of increase in the platelet count in group A was larger than that in group B (Fig. 1a, middle and right panels). In group A, 87.0% (20/23) of the patients showed a platelet increase of ≥ 20,000/µL. In contrast, only 25.0% (2/8) of the patients in group B showed a platelet increase of ≥ 20,000/µL (Table 1). The days required to reach the maximum platelet counts did not differ between groups A and B. Among 8 patients in group B, 7 received platelet transfusions due to a low platelet count of < 50,000/µL, even after lusutrombopag treatment (one patient failed to receive a platelet transfusion). However, the increase in the platelet count after platelet transfusion was not statistically significant (Fig. 1b).

### Factors that interfered with the achievement of a platelet count of ≥ 50,000 on lusutrombopag treatment

We identified factors that interfered with the achievement of a platelet count of ≥ 50,000/µL after the initiation of lusutrombopag. A low platelet count and a high splenic volume were identified as associated factors in a univariate analysis (Table 2). A multivariate analysis demonstrated that a low platelet count at baseline was significantly associated with failure to achieve a platelet count of ≥ 50,000/µL (Table 2). Indeed, all patients with a platelet count of < 30,000/µL failed to achieve a platelet count of ≥ 50,000/µL after lusutrombopag treatment. Although we tried to identify the factors that contribute to increase of platelet count ≥ 20,000 /µL from the baseline, no such factors, including platelet count at baseline and splenic volume, were identified in the univariate or multivariate analyses (data not shown).

**Table 2:** Factors that interfere with platelet count ≥ 50,000/μL

	Univariate analysis		Multivariate analysis	
	OR (95% CI)	p-value	OR (95% CI)	p-value
Age	1.01 (0.92–1.10)	0.873		
Child–Pugh score	1.07 (0.58–1.95)	0.837		
ALBI	3.70 (0.79–17.4)	0.098	2.97 (0.44–19.8)	0.26
FIB-4	0.99 (0.83–1.19)	0.949		
Platelet count	12.8 (2.01–81.4)	0.007	11.4 (1.21–107)	0.034
Splenic volume	1.00 (1.00–1.00)	0.040	1.0 (1.00–1.00)	0.72

*ALBI* albumin-bilirubin, *FIB-4* fibrosis-4

### Repeated use of lusutrombopag produced the initial response

**Fig. 2:**
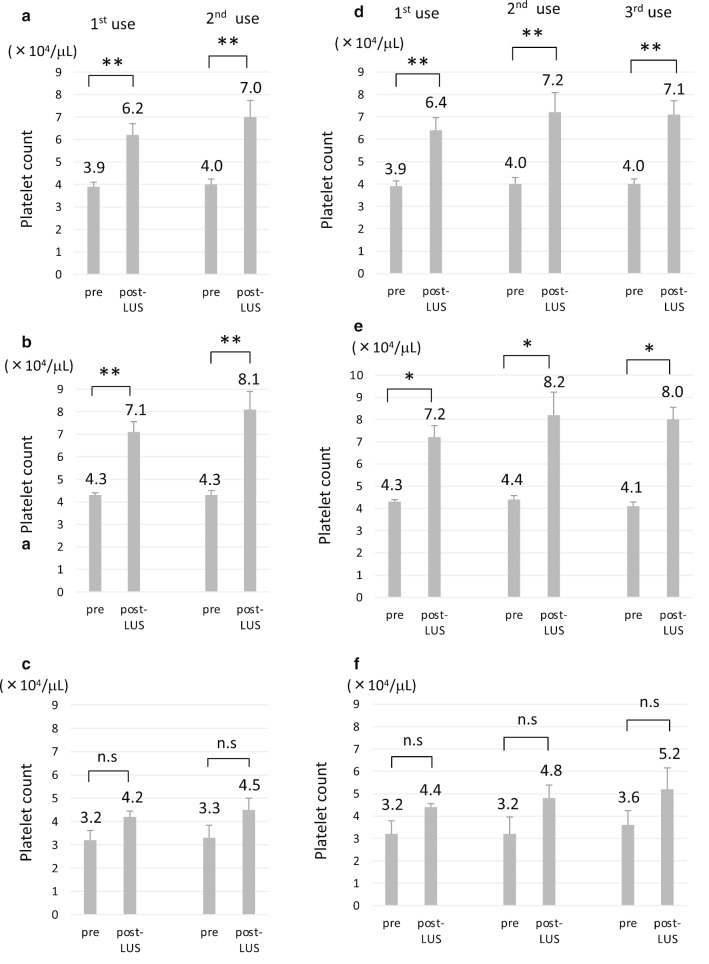
The platelet count in patients who received repeated lusutrombopag treatment (n = 13). **a**–**c** The platelet count in patients who used lusutrombopag 2 or more times in all patients (n = 13) (**a**), group A (n = 9) (**b**), and group B (n = 4) (**c**). **d**–**f** The platelet count in patients who used lusutrombopag 3 or more times in all patients (n = 10) (**d**), group A (n = 7) (**e**), and group B (n = 3) (**f**). *p < 0.05, **p < 0.01. *n.s* not significant, *LUS* lusutrombopag

Because multiple invasive procedures were often needed for patients with liver cirrhosis, lusutrombopag was repeatedly used in 13 patients; 10 patients of these patients received lusutrombopag 3 or more times. The maximum use was 5 times in one patient. The intervals of the use ranged from 35 to 781 days (median 166 days). The 2nd use of lusutrombopag significantly increased the platelet count in all 13 patients (p < 0.01) (Fig. 2a) and group A (p < 0.01) (Fig. 2b). The response to lusutrombopag in group B was similar to the initial response (Fig. 2c). Although the maximum platelet count tended to be higher in the 2nd treatment in group A, the difference was not statistically significant. We also investigated 10 patients who received lusutrombopag 3 or more times. In 10 patients, the response to the 3rd treatment was similar to that to the 2nd treatment (Fig. 2d); this was observed in groups A (Fig. 2e) and B (Fig. 2f). Of note, the mean platelet count was > 50,000 /µL at the 3rd treatment in group B, which was in contrast to the initial and 2nd treatments (Fig. 2f). In 4 patients who received lusutrombopag 4 or more times, the 4th and 5th treatments increased the platelet count as much as the initial treatment (data not shown). When all repeated uses of lusutrombopag were counted among these 13 patients, platelet transfusion was not required in 82.1% (23/28) of treatments. Patients in group A did not require platelet transfusion in their repeated uses of lusutrombopag. In addition, 50% (5/10 treatments) did not require platelet transfusion in group B.

### Sustained virologic response (SVR) increased baseline platelet count in patient with hepatitis C virus (HCV) infection

**Fig. 3:**
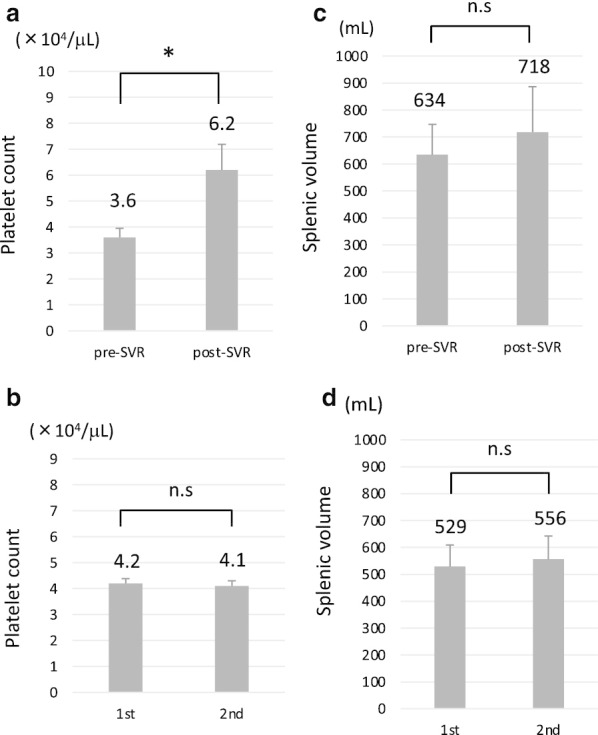
The platelet count (**a**, **b**) and splenic volume (**c**, **d**) in patients with chronic HCV infection. Patients who achieved an SVR with antiviral treatment (**a**, **c**) (n = 7). Patients who did not achieve an SVR (**b**, **d**) (n = 8). p < 0.05, *n.s* not significant. SVR, sustained virologic response

In one patient in group B, the platelet count restored to ≥ 50,000/µL following lusutrombopag treatment after the successful eradication of HCV, which was determined by SVR for 24 weeks after the end of treatment (a combination of direct acting antivirals); although the first administration of lusutrombopag soon after an SVR failed to increase the platelet count to ≥ 50,000/µL, the 2nd to 5th treatments succeeded in increasing platelet count to ≥ 50,000/µL. Thus, we investigated the effect of HCV infection on platelet counts. Among 15 patients with chronic HCV infection, 7 received lusutrombopag after achieving an SVR. We noted that 2 patients showed a platelet count of ≥ 50,000/µL at baseline after achieving an SVR. Because the platelet count at baseline was a factor that predicted the response to lusutrombopag (Table 2), we compared the platelet count at baseline and the splenic volume before and after achieving an SVR. The platelet count at baseline significantly increased after achieving an SVR (p < 0.05) (Fig. 3a). In contrast, the platelet count in the non-SVR group tended to decrease during the study period (Fig. 3b). The splenic volume was not associated with the SVR (Fig. 3c, d).

### Safety of lusutrombopag

One patient showed portal thrombosis after lusutrombopag treatment. The platelet count was 63,000/µL when the thrombosis was noted at the procedure. However, the thrombosis disappeared after treatment with an antithrombin III product for 5 days and pivaroxaban for 30 days. Lusutrombopag was well tolerated and no symptomatic adverse effects were observed during its administration of lusutrombopag. No hemorrhagic complications occurred during or after the procedure in any group.

### The effect of platelet transfusion on the increase of the platelet count

**Fig. 4:**
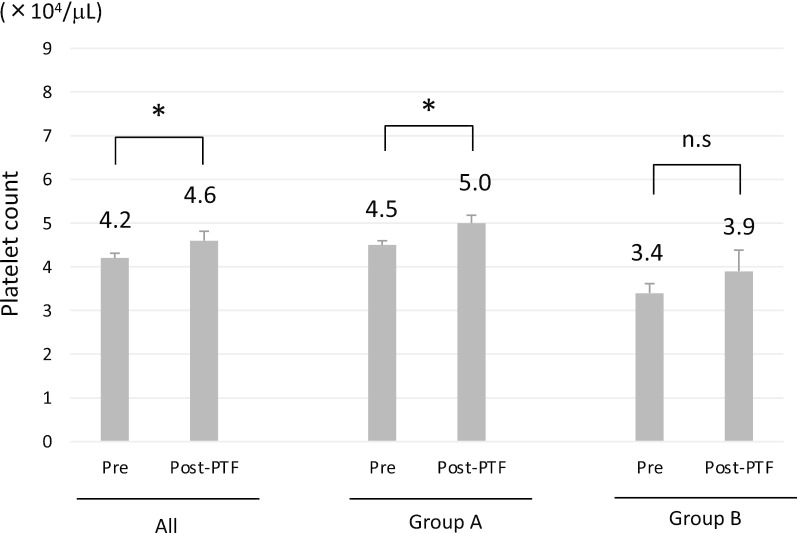
The effect of platelet transfusion on the platelet count before lusutrombopag was available (all, n = 15, left panel, group A, n = 10, group B, n = 5). *p < 0.05, *n.s* not significant, *PTF* platelet transfusion

Finally, we investigated the effects of platelet transfusion in the past. Among 31 patients enrolled in the present study, 15 patients (48.4%) had experienced platelet transfusion due to platelet count of < 50,000/µL before lusutrombopag was approved. Platelet transfusion was performed approximately 3 years before lusutrombopag treatment. Although platelet transfusion significantly increased the platelet count in comparison to baseline (p < 0.05), the mean increase was 4,000/µL (Fig. 4, left panel). There were 10 and 5 patients who had a history of platelet transfusion in groups A and B, respectively. The increase in the platelet count by platelet transfusion was small in both groups (Fig. 4, middle and right panels). Among the 15 patients underwent platelet transfusion in the past, 10 patients (66.7%) could proceed to invasive procedures without platelet transfusion after lusutrombopag treatment. These data indicate that the ability of platelet transfusion to increase the platelet count is limited in patients with platelet counts of < 50,000/µL. In addition, platelet transfusion in the past did not influence to the effect of lusutrombopag.

## Discussion

In the present study, 74.2% of patients who received lusutrombopag treatment could avoid platelet transfusion before invasive procedures. In addition, the repeated use of lusutrombopag showed similar efficacy to the initial treatment. Furthermore, no serious adverse events were observed during or after lusutrombopag treatment. Thus, lusutrombopag was considered effective and safe for CLD patients with a platelet count of < 50,000/µL.

Although platelet transfusion has traditionally performed to increase platelet counts, we have little information on the platelet count increase in patients with liver cirrhosis. Tripodi et al. reported that platelet transfusion increased the peripheral platelet count by 13,000/µL in patients with liver cirrhosis [19]. In the present study, the increase in platelet was 4,000/µL by platelet transfusion before lusutrombopag became available. One of the differences between Tripodi’s study and our study was the time point at which platelets were counted. Tripodi et al. performed the count 1 h after platelet transfusion whereas we did it on the next day after platelet transfusion. In addition, the amounts of platelets transfused differed, with ≥ 3 × 10^11^ platelets in Tripodi’s study and ≥ 2 × 10^11^ platelets transfused in our study. Hirooka et al. reported that only 5% (1/20) patients who received 10 units (≥ 2 × 10^11^) of platelets showed a platelet count of ≥ 50,000/µL [20]. These data indicate that it is difficult to increase number of platelets to a sufficient level by standard platelet transfusion in patients with liver cirrhosis.

Real-world data showed that 84–96% of patients could avoid platelet transfusion in Japan [11–13]. However, these studies included patients with a platelet count of > 50,000/µL at baseline. Because the characteristics of patients were reported to be a low platelet count at baseline in whom lusutrombopag failed to increase a platelet count of > 50,000/µL [11, 20], it is reasonable that our study showed a relatively low rate in avoiding platelet transfusion. In clinical trials that restricted enrollment to patients with a platelet count of < 50,000/µL at baseline, 72.5–79.2% of patients avoided platelet transfusion. In addition, 14.6–34.1% of patients had a platelet count of < 35,000/µL at baseline [8, 9]. The present study restricted the enrolled patients to those with a platelet count of < 50,000/µL at baseline; 74.2% of the patients avoided platelet transfusion and 29.0% of the patients had a platelet count of < 35,000/µL at baseline. Thus, the present study reproduced the results of clinical studies in the real-world setting.

Our study included 13 patients who used lusutrombopag two or more times, and the repeated use of lusutrombopag was found to be effective and safe. Although the 2nd use of lusutrombopag tended to increase the platelet count in comparison to the first treatment, this may depend on the day when platelets were counted. In the first lusutrombopag treatment between 2016 and 2017, we performed invasive procedures at approximately 8–12 days after the initiation of lusutrombopag treatment. In the repeated use of lusutrombopag after 2018, the procedures were performed at approximately 13–18 days after the initiation of lusutrombopag. This suggests that the platelet count was determined before it reached the maximum level in the earlier cases (2016–2017).

We noted that the 3rd lusutrombopag treatment increased mean platelet count to ≥ 50,000/µL in group B, in which platelet count was < 50,000/µL at the first lusutrombopag treatment. There were patients with chronic HCV infection who achieved an SVR in group B. Indeed, the baseline platelet count was significantly elevated in 7 patients after the achievement of an SVR in both groups A and B. Ishizu et al. reported that an SVR can increase the platelet count by reducing the splenic volume [21]. However, the mean splenic volume did not decrease, even after the achievement of an SVR in the present study. In fact, the splenic volume increased in 71.4% (5/7) of the patients who had achieved SVR in the present study, with the exception of two patients with relatively small splenic volumes. This discrepancy may have been due to the difference in the splenic volume at baseline. The medians splenic volume in the present study was 656 mL, while that in Ishizu’s study was 242 mL [21]. Thus, other mechanisms accounted for the increased platelet count in group B patients who received repeated treatment. There are several mechanisms by which HCV infection reduces platelet count, including bone marrow suppression and immune dysfunction [22]. In fact, patients with a non-SVR tended to show decreased platelet count during the study period. Although we did not examine detail mechanisms related to HCV infection in the present study, the status of HCV infection seemed to alter the response to lusutrombopag.

Although one patient showed portal thrombosis after lusutrombopag treatment, the thrombosis was disappeared by anticoagulant therapy. Because the maximum platelet count after lusutrombopag treatment was 63,000/µL in that patient, we considered coagulopathy related to liver cirrhosis also contributed to the development of portal thrombosis. However, we have to pay attention to the portal thrombosis.

## Conclusion

The proportion of patients who did not require platelet transfusion was 74.2%, which is smaller than that in former studies which included CLD patients with a platelet count of > 50,000/µL. However, lusutrombopag is effective and safe for CLD patients with a platelet count of < 50,000/µL. Thus, using lusutrombopag, we can perform invasive procedures without platelet transfusion in the majority of patients with severe thrombocytopenia.

## Supplementary information

**Additional file 1.Table S1.** Data set of the present study

## Data Availability

All datasets used and analyzed in the present study are available in Additional file 1: Table S1.

## Abbreviations

CLD: Chronic liver disease
CT: Computed tomography
ALBI: Albumin–bilirubin
FIB-4: Fibrosis-4
HCV: Hepatitis C virus
SVR: Sustained virologic response
